# “It’s a kind of freedom”: adolescents and parents speak about motivations for active travel and COVID-19

**DOI:** 10.1080/17482631.2022.2130508

**Published:** 2022-10-13

**Authors:** Sharon Levi, Orna Baron-Epel

**Affiliations:** School of Public Health, University of Haifa, Haifa, Israel

**Keywords:** Active travel, physical activity, adolescent-parent dyads, COVID-19, qualitative interviews

## Abstract

**Purpose:**

Active travel (AT) incorporates physical activity into daily living, critical for healthy adolescent development. We explore adolescent and parent attitudes and behaviours related to motivations for adolescent AT and effects of the COVID-19 pandemic.

**Methods:**

We conducted semi-structured Zoom interviews with 25 adolescent-parent dyads in communities across Israel during early stages of the COVID-19 pandemic. Thematic content analysis was used to develop categories and themes.

**Results:**

We identified key themes related to adolescent AT: Fostering independence enables adolescent AT; Pampering and safety concerns inhibit adolescent AT; Family and community norms influence adolescent travel modes; Personal enjoyment and positive attitudes facilitate AT; Peers and social networks promote adolescent AT and PA; Built environment and transport options influence AT choices. Interestingly, adolescents indicate AT is an opportunity for peer-to-peer communication without screen distraction, yet they use social media to promote AT and PA.

**Conclusions:**

The findings point to the influence of positive parent perceptions, active and supportive family and community norms on adolescent AT. Peer norms and social networks as well as features of the built environment also have the potential to influence AT. The COVID-19 pandemic encouraged use of AT and provided a setting for positive AT experiences.

## 1. Introduction

Adolescence is a critical period of transition from childhood to adulthood, characterized by rapid physical, neurological, psychological, and social development. Issues related to wellbeing that arise during adolescence, such as obesity and sedentary behaviours, are related to future disease burden and public health (Alberga et al., 2012). Consequently, adolescence is a significant time to establish lifestyle behaviours that will promote both present and future health (Craigie et al., 2011). Regular participation in physical activity (PA) is an important behaviour related to long-term physical and mental health (Biddle et al., 2019; Janssen & LeBlanc, 2010). However, globally levels of PA are low, with four in five adolescents not achieving the minimum recommendations of 60 minutes or more of daily moderate to vigorous PA (WHO Regional Office for Europe, 2018).

Active travel (AT), including walking and bicycling, is one method to incorporate PA into daily living. At the individual level, AT is related to increased PA and physical fitness for adolescents (Henriques-Neto et al., 2020). At the community level, increasing AT can support social connections and environmental health; a shift from motorized travel to AT supports policy efforts worldwide on behalf of sustainable development and contributes to lower carbon emissions in the built environment (Brand et al., 2021; OECD, 2017).

Parents and parental support play an important role as regards adolescent PA with parental encouragement, logistical support, and co-participation supporting these activities (Edwardson & Gorely, 2010; Rhodes et al., 2020; Sallis et al., 2000). Previous research, primarily focused on travel to school, indicates that perceptions related to the environment and social support, from peers and parents, are associated with rates of AT among adolescents (Ikeda et al., 2018). Additional key psychosocial factors related to increased AT, include self-efficacy and enjoyment of PA (Wang et al., 2017). Social interactions in the context of AT are also associated with increased mobility; for adolescents there is an emphasis on AT with peers (Panter et al., 2008). Correspondingly, a series of focus groups, conducted with children and adolescents following implementation of several initiatives to promote active school travel in Auckland, New Zealand, found that enjoyment was an important factor for both age groups, travelling with friends and having fun (Hinckson, 2016). As concerns the built environment, improved infrastructure for walking or bicycling and creating safe places to walk were found to have a significant impact on rates of child AT (Smith et al., 2017). Concurrently, parent perceptions regarding a lack of safety, including dangers of traffic and crime in diverse environments, have been found to be associated with lower rates of PA, lower AT and independent mobility, and may result in restrictions on adolescent AT (Carver et al., 2010; Esteban-Cornejo et al., 2016; Riazi et al., 2019). Similarly, in a mixed methods study with grade school children, parents and school representatives regarding active school travel utilizing a shared path in Whangarei, New Zealand, parents indicated that traffic and safety concerns influence choice of travel mode.

Israel is considered a densely populated country; in 2018, 92.4% of the Israeli population lived in urban areas, compared to 78.7% in other more developed regions in the world (United Nations, 2018). Recently, there has been an increased emphasis on design of urban facilities to support AT by the Ministry of Transport, however bicycle lanes and paths in particular are not common yet in most Israeli towns, and walkability in many communities is inadequate (Gitelman et al., 2020; Levi et al., 2015). Simultaneously, there is evidence that children and adolescents are vulnerable road users in urban areas in Israel; an analysis of pedestrian crash data, revealed that the share of severe injuries among children was consistently higher than among adults, by 1.25 times, on average (Levi et al., 2015). Moreover, in a study of naturalistic behaviours for a large population of children and adolescents at urban intersections, risk-taking behaviours were higher for adolescents, including crossing on red, while distracted and without checking traffic (Gitelman et al., 2019). Previous research on child and adolescent AT in Israel is limited and has primarily focused on specific urban areas. A study on travel patterns in three cities in Israel found that children and adolescents are active and independent travellers, along various routes throughout the day utilizing walking for the majority of their trips (Levi et al., 2015). Another study with 5th and 6th graders in a large municipality in central Israel found that higher rates of walking were identified in the more highly urbanized areas; however, cycling was negatively related to highly urbanized areas with multiple intersections and residential density (Moran et al., 2016). Issues related to safety, convenience, facilities, and comfort all have the potential to affect attitudes of both adolescents and parents to use of AT.

The outbreak of COVID-19 resulted in numerous restrictions related to additional reductions in PA and increases in sedentary activities. Public health professionals have voiced concerns that behaviours during COVID-19 have the potential to further impact and accelerate the pandemic associated with the lack of PA and sedentary behaviours related to chronic disease (Hall et al., 2021). In Israel, the response to COVID-19 included closure of secondary schools, cancellation of youth activities, limits in distances for walking and bicycling, and shut-down of both private and public facilities and organizations that serve as focal points for adolescent PA and AT. Pursuant to this, findings of a survey on household health behaviours in Israel during COVID-19 indicate that among respondents with children under age 18, 65% reported a decrease in their children’s PA in both continuation and frequency (Laron & Goldwag, 2020).

Previous research on facilitators and barriers to AT has primarily focused on active school travel, with limited qualitative insights on how adolescents and parents coalesce their similar or different points of view regarding this behaviour. This qualitative study was conducted with adolescent-parent dyads to better understand adolescent and parent attitudes and behaviours to motivations for adolescent AT for both school and leisure purposes. In addition, the study seeks to assess the effects of the COVID-19 pandemic on adolescent AT behaviours and attitudes.

## 2. Methods

### 2.1 Participants

Purposive sampling was conducted to identify Hebrew speaking households, with both an adolescent age 13 up to 17 (driving age) years and parent in residence. The sample included representation of different types of communities, including urban, suburban, and rural settings across Israel. Exclusion criteria included adolescents or parents not living at home full time.

A prepared recruitment message was sent via WhatsApp to groups and individuals requesting participation in interviews on adolescent travel behaviour conducted in the framework of PhD research. Respondents to the message were contacted via telephone to describe the study. Letters of consent for the parent and adolescent were sent in advance. Consent was verified orally at the start of the interviews by both the parent and adolescent. Following initial telephone conversations and informed consent there were no refusals; there were two households with which there were difficulties scheduling an appointment and ultimately after multiple attempts these households were not included in the final sample. The study was approved by the Ethics Committee—Institutional Review Board governing the use of Human Participants in Research, Faculty of Social Welfare & Health Sciences, University of Haifa (No. 087/20).

As the study progressed, the sample was augmented for a variety of households and to attempt to ensure theoretical saturation of the topic. Analysis of interviews identified additional topics for integration in the guide, to obtain wider and significant information in subsequent interviews. Once no supplementary topics were revealed, by code as well as by meaning, data saturation was reached and further data became superfluous (Hennink et al., 2017). This process determined the sample size and saturation was achieved after 25 adolescent-parent dyads were interviewed. Most participants expressed themselves in a detailed manner, following the 25 pairs new or more in-depth knowledge no longer emerged and saturation was attained.

### 2.2 Interviews

A semi-structured interview format was used for both the adolescent and parent, with open-ended questions to elicit information on different topics related to adolescent and parent AT and PA behaviours, perceived facilitators and barriers to use of AT as well as effects of COVID-19 (Appendix A). In addition to general questions on motivations for use of AT modes, probing questions were utilized to further investigate issues such as the effects of peers, social and community norms, and the environment. The interview with adolescents includes completion of a trip diary tool to record trips during a full day (Levi et al., 2015; Stark et al., 2015). Demographic data were collected on age, gender, household income, vehicle and bicycle ownership, and parent education status. The questions were used as cues to help guide and focus the interview; however, the questions were tailored to each situation. The guide was developed by the research team and reviewed with two adolescents and two parents prior to commencement of the interviews.

The interviews were conducted during the COVID-19 outbreak, lockdown, and initial return to routine schedules in Israel (March–June 2020; Ginsburg, 2020). Due to the restrictions, interviews were conducted via Zoom digital platform. Back-to-back interviews of the adolescent, followed by the parent, were scheduled at a time that was convenient for the household, typically in the late afternoon or evening. Interviews with the adolescent were 30–45 minutes and interviews with parents were 20–30 minutes. The interviews were conducted separately, in a few cases parents stayed in the room for the beginning of the interview with their child, to ensure comfort with the discussion. The adolescents did not participate in the parent interview. The interviews were audio-recorded on a separate recorder and then transcribed verbatim. Field notes were captured immediately following the interview. No repeat interviews were conducted, and the participants were not contacted following the interviews to share transcripts or request feedback on the analysis.

The interviews were conducted by a researcher with previous experience and training in conducting interviews and focus groups with adolescents, parents, adults, and professionals on related topics including child and traffic safety, travel, and health behaviours. During the study, the researcher was engaged in PhD research at the School of Public Health, University of Haifa and as a researcher in a National Program for Active & Healthy Living at the Ministry of Health. Participants were informed in advance that the research was conducted as a component of PhD studies. During the interviews with adolescents and parents, the interviewer indicated that the topic is of both personal and professional interest.

### 2.3 Qualitative analysis

A systematic approach for the qualitative data analysis was used based on the categories represented in the interview guide, as well as an inductive approach in which new categories were developed as analysis of the findings progress (Corbin & Strauss, 1990; Gioia et al., 2013). Data familiarization, coding, identification of patterns, and theme development were conducted by one member of the research team using ATLAS.ti 8 software. The steps included open coding based on review of all interview scripts to determine a preliminary structure of codes and categories, followed by axial coding in which relationships were identified between the different categories and development of initial themes based on the patterns identified. Following coding of the first five interviews, the two-person research team met to review the codes and categories in detail and to discuss additional concepts, and this was followed by additional consultation to deliberate and refine key categories and emerging themes. The process was recursive as delineated in the reflexive thematic analysis process, with multiple returns to the data set and detailed review, analysis, and development of the themes (Braun & Clarke, 2021). Based on the coding and analysis phases, central themes were identified, supported by multiple subthemes, as detailed in the results section.

## 3. Results

### 3.1 Demographics

The interviews were conducted with 25 adolescents and 25 parents, one parent for each teen. Table 1 provides details about the household and participant characteristics for each of the participants. Overall, the rates of PA and AT were higher for adolescents than for parents.

**Table 1.** Adolescent–parent dyad interviews, household and participant characteristics.

Household (N = 25)			
Income^1^	Below Average	3	(12%)
	Average	3	(12%)
	Above Average	19	(76%)
Vehicle Ownership	1	8	(32%)
	2	17	(68%)
Urbanicity	Cities	16	(64%)
	Towns and Suburbs	7	(28%)
	Rural Area	2	(8%)
Stage of COVID-19	Lockdown	8	(32%)
Restrictions	Easing of Restrictions	8	(32%)
	Return to Routine	9	(36%)
Adolescent (N = 25)			
Age Group	13–14	10	(40%)
	15–16	15	(60%)
Gender	Male	10	(40%)
	Female	15	(60%)
Physical Activity^2,3^	0–1 times a week	7	(28%)
	2–3 times a week	6	(24%)
	4+ times a week	12	(48%)
Active Travel^4^	0–1 trips a week	1	(4%)
	2–3 trips a week	8	(32%)
	4+ trips a week	16	(64%)
Distance to School	Up to 2 Km	8	(32%)
	More than 2 Km	11	(44%)
	In another town	6	(24%)
Bicycle Owners	Yes	14	(56%)
	No	11	(44%)
Parent (N = 25)			
Physical Activity^2^	0–1 times a week	9	(36%)
	2–3 times a week	10	(40%)
	4+ times a week	6	(24%)
Active Travel^4^	0–1 trips a week	13	(52%)
	2–3 trips a week	6	(24%)
	4+ trips a week	6	(24%)
Education	High School/ Vocational	6	(24%)
	College Degree	9	(36%)
	Post-Graduate	10	(40%)

^1^The question indicated average household income of 17,300 NIS, based on data from the Central Bureau of Statistics 2020, and asked if household income is average, above average, below average.

^2^Number of times a week PA at least 30 minutes.

^3^PA in addition to school physical education.

^4^Number of times a week of AT for more than 10 minutes.

### 3.2 Themes

Participants, adolescents, and parents discussed the different facilitators and barriers to adolescent AT. The analysis revealed key themes related to motivations for adolescent AT: Fostering independence enables adolescent AT; Pampering and safety concerns inhibit adolescent AT; Family and community norms influence adolescent travel modes; Personal enjoyment and positive attitudes facilitate AT; Peers and social networks promote adolescent AT and PA; Built environment and transport options influence AT choices. Each of the themes was supported by multiple subthemes (see Table 2). Furthermore, the effects of COVID-19 as well as proposed interventions related to respondents were identified in relation to each of the key themes.

**Table 2.** Key themes, subthemes, and effects of COVID-19, and proposed interventions related to adolescent active travel.

Themes	Subthemes	Effects of COVID-19	Proposed Interventions and Messaging
Fostering independence enables adolescent AT	• Support for independence and development of skills facilitates adolescent AT • Perception of health benefits by parents relates to promotion of AT	COVID-19 increased view of AT as freedom	Emphasis on skills gained using AT
Pampering and safety concerns inhibit adolescent AT	• Parents coddling decreases use of AT • Concerns regarding both personal and traffic safety are barriers to AT	Fears related to COVID-19, in particular in public transit, led to increased use of private vehicles	Programs that encourage parents to let go, encourage independence
Family and community norms influence adolescent travel modes	• Family norms of AT and PA facilitate AT • Communities with ATS programmes and equipment encourage AT • Common use of personal vehicles and carpooling in the community is a barrier to AT	COVID-19 facilitated further AT and PA with family and in the community	Programming that encourages joint AT with parents. City and school AT programs
Personal enjoyment and positive attitudes facilitate AT	• AT and PA are fun and feel good • AT is time for oneself • Use of AT is a personal choice	COVID-19 increased personal use of AT	Emphasis on the fact that AT feels good, rather than on health
Peers and social networks promote adolescent AT and PA	• AT is preferred with peers • AT is good for peer-to-peer communication • Social networks are used to encourage AT or PA	COVID-19 increased use of AT and PA to be with friends	Programs that promote AT with peers And use social networks to promote AT
Built environment and transport options influence AT choices	• Proximate destinations encourage use of AT • Facilities including bicycling paths, sidewalks, shade, and pleasing environment facilitate use of AT • Availability of public transit supports use of AT as part of the trip • Difficult topography, poor facilities, distances are barriers to AT	COVID-19 emphasis on accessible and pleasing AT facilities.	Improve AT and PA facilities in the environment

While both parents and adolescents spoke to facilitators and barriers to AT associated with each of the key themes, the first three were found to be directly related to the parental attitudes and behaviours and the family structure. These are followed by themes related to the effects of personal attitudes, peers, and the environment on AT choices made by adolescents.

#### 3.2.1 Fostering independence enables adolescent AT

Adolescents indicated that AT is an opportunity for independence, as one voiced, “You are more dependent on yourself and they do not tell you ‘At this hour and this I can take you and at this hour and this I can bring you back’. You can go and come back whenever you want.” Many parents also point to independence as an important benefit, in particular those whose children were more frequent AT users, as one mother indicated, “I think it’s part of growing up and part of being in the real world. Being independent not relying on people to drive you everywhere.” Concurrently, these same parents also pointed to other advantages, which the adolescents did not voice, including acquiring skills such as navigation and time management as well as personal empowerment and self-esteem. One mother shared about her son, “He is independent. He operates on his own, makes his plans, isn’t dependent on when Mom can drive him … [a ride] becomes a resource that needs to be managed and I see he manages this resource very seriously.” Highlighting these skills was mentioned as a method to increase awareness of the benefits of adolescent AT.

A notable distinction between adolescent and parent perceptions related to the relationship between AT and health and PA. Adolescents generally did not point to health benefits of AT. Adolescents were more likely to count AT as PA if they used walking, running, or bicycling purposefully for PA, otherwise AT was primarily viewed as a travel mode. As one voiced, “I would not consider it unless I walk a lot that day. For example, if I walk from the shopping center to my house, that has happened several times, it’s a lot of walking. An hour or more of walking. But I wouldn’t count just walking around town [as PA].” In contrast, parents were far more likely to indicate that a major benefit of AT is that it is healthy, a form of PA, for themselves as well as for their children, even in cases in which their child is already participating in organized PA. While parents voiced the health benefits of AT, we found that the importance of fostering independence was more central and related to both parent and adolescent attitudes and motivations for AT.

In COVID-19, the importance of adolescent AT for increased independence was emphasized. In the earlier stages of the lockdown opportunities for AT were limited, adolescents voiced their frustration. As restrictions eased, renewed, and increased opportunities for AT were related to freedom and independence. As one adolescent stated, “Before Corona I rarely rode my bike, only once in a while, but during Corona […] I think it pretty much opened up this opportunity for me […] it’s a kind of freedom and I ride a lot on a bike.” Parents also pointed to further use of adolescent AT and PA during the pandemic for independence as well as time outdoors with peers.

#### 3.2.2 Pampering and safety concerns inhibit adolescent AT

Parent attitudes that support a desire to cosset or pamper were found to impede use of AT and increase provision of rides by personal vehicle. One mother described this as, “we spoil them, wrapping them in cotton wool.” Parents related that provision of rides often becomes a routine. A mother shared, “I don’t know, it could be a habit, we got used to it and it’s part of our routine in the afternoon, it’s as if it is a part of parenting.” We found that parents related this behaviour as a continuation from earlier childhood, and often for these parents this attitude was in lieu of promoting adolescent independence. Similarly, this attitude was adopted by some adolescents; provision of rides is expected even to nearby destinations. Along these lines, parents indicated that an AT intervention programme that provides a framework to promote increased adolescent freedom and independence should be encouraged; our understanding was that they viewed this as an opportunity to transition to more autonomous adolescent behaviour.

Both parents and adolescents voiced fears related to traffic and personal safety that impact use of AT. Issues raised include vehicles that do not yield right-of-way, lack of safe bicycling paths, night, and wildlife. The fears voiced by adolescents were often repeated by parents aware of their child’s concerns, as one mother shared, “There’s an area right next to the house when he arrives he turns on a flashlight. It’s a ritual. He calls and we go out to meet him. If possible we drive to pick him up from the sports field. Again, if it’s in the evening. He is terribly afraid of the wild boars […] in the neighborhood.” In some cases, parents were more likely to voice reservations to AT than their children. One example is related to bicycling and e-bicycling, which was often viewed more favourably by adolescents than parents. One mother related her fears, “I’ll tell you the truth, I’m not so happy about a bike. I believe it is very dangerous […] I prefer to drive them. I remember as a girl the amount of child accidents on bicycles, so I’m just not ready.”

COVID-19 created new reservations regarding travel, for both parents and adolescents in particular, as regards use of public transport. In some cases, fears that adolescents expressed regarding potential contagion were stronger than their parents, while others voiced the fears that they heard from their parents. One adolescent shared, “Now in COVID […] I do not want to take a bus because it feels dangerous to me in terms of getting infected. My Dad told me not to take the bus, he told me to take a bike.” However, others related that the situation increased their use of personal vehicles, as related by another adolescent, “I am not allowed to travel by public transport at the moment. So, my parents pick me up and drop me off from school and wherever I need to go.” Overall, participants voiced that the initial phases of the pandemic increased dependence of adolescents on their parents.

#### 3.2.3 Family and community norms influence adolescent travel modes

Parents and adolescents spoke about community norms that promote use of personal vehicles, as a barrier to adolescent AT, and these in turn often served as the basis for family norms. These were often suburban environments in which the common mode of transport for adolescents was via carpooling. As one mother shared, “Most of the trips are by vehicle, those who don’t are parents who aren’t available, most of the parents I know give rides. Once when there was practice in the afternoon and no mom was available to drive them they took the bus or walked, but when it is possible we drive them.” It is interesting to note that suburban environments in Israel are relatively dense (towns); many offer access to high-quality sidewalks and parks, with a variety of distances and fewer public transit options. In some cases, these norms were also reflective of higher socio-economic circumstances, however this was not always the case. We found that these community norms were habit forming, parents viewed provision of rides as standard behaviour but did not usually reflect resistance to the use of AT. It is possible that by promoting different values in combination with AT such as joint peer activity, health, or environment consciousness it may be possible to develop new norms in these communities.

Correspondingly, several parents and adolescents pointed to norms that promote AT use in the community and in their family. Communities with walk-to-school programmes (primarily in grade school) and installation of bicycle racks were viewed as conducive to use of AT. In urban areas many participants pointed to common use of AT in the community, an adolescent shared, “As far as I am concerned we walk. The whole neighborhood here has to walk to the mall, to school, in our experience we are walking.” Both parents and adolescents spoke to the potential of intervention programmes that increase AT norms, in schools and throughout the community, including specific inducements such as adding bicycle racks or providing incentives.

We also heard from many parents and their children, similar patterns of behaviour and family norms that promote AT and PA. One mother shared, “As a mother it’s part of our education, from a young age they are connected to PA, there is always a sports class and they like to move. We never had, or allow, electric ATVs or bikes. It’s a principle that accompanies us, that from a young age you tell them it’s much more important [to use AT], healthier. Both healthy for you and much more environmentally friendly.” We did not find that these types of parents or households were representative of a specific demographic or geographic context. Moreover, COVID-19 offered new opportunities for joint AT and PA with the nuclear family. In particular, the second stage, when there was an easing of lockdown. One adolescent stated, “During Corona I actually took some walks with mom and some runs with dad … we took advantage of it. Usually, we don’t really do such things [because we use the vehicle].” Several adolescents and parents indicated that these occasions were viewed as quality time, for example, one mother stated, “Yes in general the Corona was an amazing time […] I do not remember a time that was so cohesive and unifying. We did not always all go out, but we did go for walks and we said this is the opportunity to get fresh air. We did not call it sports.” Similarly, some participants shared that during the pandemic they saw considerably more AT users in the community.

#### 3.2.4 Personal enjoyment and positive attitudes facilitate AT

Adolescents voiced that personal enjoyment is an important motivation for AT as well as PA. Rather than speaking to health benefits as was more common among parents, adolescents who utilize AT regularly were likely to indicate that it is fun and feels good. In contrast, adolescents indicated that negative circumstances such as intense heat or heavy rain reduce the enjoyment and therefore serve as a barrier to AT. In line with this, in speaking about potential interventions with adolescents an emphasis is placed on the importance of speaking to the positive experience. As one adolescent shared, “First of all, saying it [AT] is healthy doesn’t convince anyone. If you tell them there is no reason not to, it’s good for your body […], helps your muscles, you’ll look good, it’s fun, and the way you feel after is worth everything.”

In addition to personal enjoyment, several adolescents indicated that they view AT as an opportunity for solitude, to listen to music and to be with oneself. One adolescent stated, “As far as walking is concerned, I think it’s nice to walk alone and think about different things, look at people around me. When I am out walking I think about different issues, and it is nice to have time to myself.” It is interesting to note that the interest in privacy may result in selection of AT over public transit throughout the year, an adolescent pointed to a preference for AT over taking the bus “I think it is comfortable for people […] to walk in the fresh air alone, it is private, you don’t have to speak to lots of people and have more personal space.”

Both adolescents and parents indicate that use of AT is usually a personal choice. While there may be some direction provided by a parent, ultimately AT use on a regular basis is self-motivated, as one mother stated, “I find that in most cases I don’t need to encourage him [to use AT], it just happens.” The COVID-19 pandemic highlighted use of AT by adolescents as an opportunity for personal enjoyment and a personal choice, use of AT alone or with peers was preferred over other shared modes of travel.

#### 3.2.5 Peers and social networks promote adolescent AT and PA

Both parents and adolescents speak of the opportunity for time with peers as a facilitator for AT as well as one of the benefits of AT. Parents often point to the social aspect of AT as one of several benefits for adolescents, as one mother stated, “Yes it’s healthy, also socially, it’s a time for her to be with friends.**”** For adolescents, time with peers was viewed as a key facilitator for AT, walking or bicycling together to a variety of destinations including both school and leisure trips. Adolescents indicate that AT with peers is a social activity and the trip is inclined to feel like it is of shorter duration, as one adolescent shared, “We usually connect transport to a destination, but when you walk with friends it is different. It is not about a particular destination it can also be a trip without a particular goal or purpose.” Use of AT with peers was also voiced as a solution to safety issues such as trips at night.

An emergent topic relates to adolescent AT in the age of social networks and high levels of youth screen time. Select adolescents voiced the specific benefit of AT, as an opportunity for higher quality personal time, without distractions and screens, for example, one adolescent stated, “Yes, because when you are walking (with a friend) you do not deal with your phone. If we sit next to each other, then we do talk but sometimes we check to see who sent a message, the time, and what it says. When you walk, you do not bother with it, you just talk and it’s fun.” Conversely, several adolescents pointed to their social networks as a method to promote AT and PA. The platforms vary, one adolescent indicated, “There is Instagram Story which everyone sees, so you know by us we post in Story, who wants to walk with me to school. And then you can write back ‘Sure I want to go, lets plan etc.’ and then we plan and go, it’s fun.” Another participant described group discussions on social networks to encourage use of AT, “Yes we plan to meet and if someone doesn’t have a way to get there, we write in the [WhatsApp] group, just get up and walk. It’s not so far that you can’t walk there, so we walk.” This duality of the contribution of AT to peer-to-peer engagement away from screen distractions while simultaneously using social media to promote AT was very interesting. We found that adolescents voiced challenges they face in conducting personal communication and it seems that AT offers a unique opportunity for engagement that is not usually available.

Importance of the peer group was further highlighted in relation to adolescent AT and PA during COVID-19. Some parents and adolescents voiced that adolescent AT and PA increased in the pandemic in order to spend time with peers, as one adolescent shared, “I’m a lazy person. I really have no energy. But yes, during the Corona it seems to have grown on me, I take an hour and set up with a friend [AT or PA]. So, it turns out that every day I made an appointment with a friend and we do it together.” In accordance with these views, parents and adolescents indicated that expanding opportunities for AT with peers is a method to increase adolescent AT overall. As one adolescent shared, “We are a screen generation, we don’t like to go out as much. Friends are more likely to visit at home. I recommend that everyone go out especially in cool weather, it is more fun and healthier. Walking is a good time to talk, it is more fun to talk while you are walking. My friends and I often will take a walk around the school campus, because it is fun to talk [while walking].” Use of social networks to increase AT was also promoted, due to the common use of the platforms and in light of the fact that adolescents indicate that they are inclined to accept invitations from a peer to use AT together.

#### 3.2.6 Built environment and transport options influence AT choices

Adolescents and parents spoke to a variety of elements in the built environment that influence use of AT modes. Proximate destinations, including schools, shops and leisure facilities, encourage use of AT, as one adolescent explained, “Yes, I don’t have a problem walking places. It’s fun, refreshing. Not walks that are too long, but I am used to it. I walk quite a lot from place to place. There are lots of places to go in the area.” Adolescents also indicated that availability of public transit options increased likelihood of using AT modes in a chained trip.

Participants pointed to a variety of facilities that improve AT conditions, including comfortable sidewalks, shade, bicycling paths, and bicycle racks at destinations. In discussing the built environment, participants referenced both accessibility and safety issues. This was emphasized for bicycling conditions, which both parents and adolescents often indicated were lacking. Several parents and adolescents stated that the lack of safe bicycling paths is a major inhibitor to use of this AT mode. In addition, participants expressed a safety concern for pedestrians, as due to a lack of safe bicycling paths many of the adolescents indicated that they ride on the sidewalks, “Yes, as far as bikes go, there aren’t enough paths. I ride on the sidewalk and I could hurt people.” One of the most common intervention methods suggested by participants across the different community settings is installation or improvement of bicycling paths, as one mother shared, “Walking is not a problem, we have pleasant sidewalks in our neighborhood, but bicycling is a problem because we have no bicycle paths and riding on the road is very dangerous.”

In addition to specific AT amenities, adolescents pointed to the fact that a pleasing environment is conducive to AT, in particular green spaces, as one participant stated, “I just got home from a walk around the neighborhood. I walk around everywhere here because it’s a beautiful town.” In contrast, an unattractive or uninteresting environment may be a barrier to use of AT. During the COVID-19 pandemic accessible and pleasant AT facilities were of particular importance, proximity to green spaces, ocean boardwalks, and other agreeable locations served as increased motivation for AT and contributed to positive attitudes regarding walking and bicycling. These facilities were accessed together with friends and family, as one adolescent commented, “During the Corona our family walked all the time together. To the beach or on the promenade … It was quality time together, walking is fun.” Efforts to improve the built environment and the addition of AT facilities were emphasized by parents and adolescents to promote increased walking and bicycling.

## 4. Discussion

**Figure 1.**
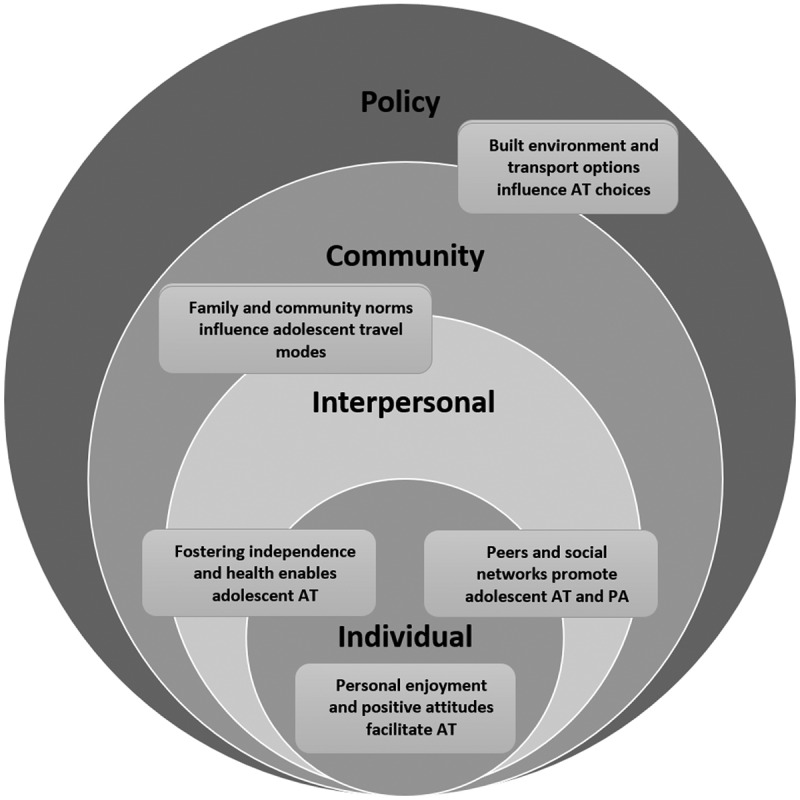
Key themes related to motivations for adolescent active travel in relation to levels of influence in the social ecological model.

Active travel is an opportunity to incorporate adolescent PA in different environments. This qualitative study investigated adolescent and parent attitudes and behaviours to AT among adolescents in Israel. Key themes were identified which also reflect different levels that support and influence health behaviours, including the individual, interpersonal, community, and policy, as expressed in the social ecological model (Mcleroy et al., 1988; See, Figure 1). In the case of AT, a study of the different levels of influence is particularly important as it has been established that multilevel interventions targeting individuals, communities, physical environment, and policy are critical to achieve behaviour change (Sallis et al., 2006; Willis et al., 2015).

In studying the key themes related to parental attitudes and behaviours and the family structure at the interpersonal and community level, we find that the information gathered on barriers and facilitators, builds on previous research on the different influences on adolescent AT. Parental views that point to an interest in fostering independence and the health advantages of AT are motivations for promoting adolescent AT. Parents also speak to self-efficacy and empowerment, in line with key psychosocial aspects previously documented in the literature (Wang et al., 2017). Both parents and adolescents emphasize the role of adolescent AT in gaining independence, which has been noted in connection with AT to primary school (Forsberg et al., 2020). We found moreover in this study that parents voiced the specific contribution of AT to life skills such as navigation and time management.

Conversely, corresponding to previous research, both parent and adolescent negative perceptions and fears related to both traffic and personal safety were found to be obstacles to adolescent AT (Esteban-Cornejo et al., 2016). In addition, we found that parents who look to pamper or “bubble-wrap” their adolescents were likely to provide more rides by private vehicle, which inhibits adolescent AT. Adolescent AT is also influenced by norms at both the family and community level. In homes and communities where use of a personal vehicle is the preferred method to transport children, adolescent AT is impeded. In contrast, family and social norms that prescribe habitual AT use have a positive influence on adolescent behaviour, similar to research on the role of parental support in increasing adolescent PA (Edwardson & Gorely, 2010; Rhodes et al., 2020; Sallis et al., 2000). We find that there is an inherent conflict for many parents between the understanding and support of the fact that AT has the potential to contribute to adolescent independence and autonomy and the ability to let go of norms and habits that encourage provision of rides. While some did indicate that there were also safety concerns that may continue to inhibit promotion of AT, for others it seems that there is potential for a shift to other alternative health promoting behaviours. Encouragement of AT behaviours relates to health promotion as well as autonomy support, which is a key factor in transition to emerging adulthood (Baumrind, 2005; Ungar, 2009). Parents may be encouraged to begin with support for AT to and from school, proximate leisure activities, and—or together with peers, while still monitoring or limiting completely independent travel in the evenings or to distant locations. Support for community or school-based AT programmes may also serve as an impetus to engage parental cooperation for adolescent AT.

Additional themes that were identified reflect direct influences on motivations for adolescent AT including individual attitudes, effects of the peer group, and factors related to the built environment. Similar to previous research, personal enjoyment was found to be a motivator for adolescent AT (Wang et al., 2017). In this study adolescents with a positive attitude towards using AT modes alone, as well as with peers and family, were likely to make a personal choice to walk or bicycle to different destinations.

The findings also contribute to the body of literature related to the importance of peer social support for AT by identifying the potential for social networks to increase awareness, positive perceptions, and promote opportunities for both AT and PA (Ikeda et al., 2018; Panter et al., 2008). Simultaneously, adolescent views regarding AT as an opportunity for peer-to-peer communication without distractions are of interest in this era in which health professionals and educators are focusing on screen time reduction efforts due to potential negative impacts on a variety of physical, cognitive, and psychosocial health outcomes (Stiglic & Viner, 2019). Future research may further explore the role of AT as an opportunity for peer-to-peer communication as well as quantitative measures of exposure to and promotion of PA and AT in social networks.

Increased walkability, connectivity, and pedestrian and bicycling accommodations have the capacity to facilitate active travel (Ikeda et al., 2018; Smith et al., 2017). Similarly, participants in the study indicated that specific environmental factors related to higher use of AT include proximate destinations and supportive infrastructure. In particular, an emphasis was made on the need for additional bicycling paths to ensure safety of all road users. At a community level, support for norms related to use of AT, minimizing vehicular traffic, and promotion of a more sustainable environment may serve to increase AT behaviours as well as compel local and national policymakers to invest further resources to improve the environment.

The findings related to the COVID-19 pandemic further highlight these key themes. Participants in the study voiced certain negative effects of COVID-19 on PA and AT during the lockdown periods similar to findings worldwide (Laron & Goldwag, 2020; Tison et al., 2020). Moreover, adolescents emphasized their frustration with the effects of the pandemic on their freedom. However, in the later phases of the pandemic, AT and PA were viewed as a method to garner independence. Additionally, both parents and adolescents voiced positive attitudes regarding the additional opportunities for joint parent and child AT and PA afforded during COVID-19. This finding together with the impact of family norms on adolescent AT underscores the importance of parent-child co-participation in support of increased AT (Rhodes et al., 2020). Active travel was also voiced as an important method to increase peer interaction during COVID-19, which may be carried through to social networks and intervention programmes to increase walking and bicycling. Finally, participants voiced the importance of pleasing and accessible AT facilities, in particular during the COVID-19 pandemic, which may serve as a unique opportunity to promote improvements to the built environment (Nurse & Dunning, 2020).

The findings of the current research should be considered in light of the study limitations. Qualitative research may be associated with information bias; therefore, questions were asked in an open-ended manner and in a range of ways to prevent the participant from simply agreeing or disagreeing. The use of a semi-structured script, with questions regarding both negative and positive perceptions and experiences, also allowed for a more open dialogue. Use of ATLAS.ti software to code each interview and map the codes across the entire set of interviews helps to maintain quality and analyse qualitative data in a methodological manner.

A further limitation is related to the context, the study was conducted in Israel. An effort was made to interview households in diverse settings in particular different levels of urbanicity, to increase transferability of the findings. To further facilitate transferability, the research process, the participants, and the results have been described in detail in the methods. It is also important to note that the participants included a higher percentage of households with above average income, a more in-depth study of lower-income households may yield additional findings.

The findings in this study with adolescent-parent dyads point to similarities and differences between parent and adolescent attitudes to AT and the important role of parents in increasing healthy behaviours. Adolescent AT may be influenced by positive parent perceptions, active encouragement of AT and PA and supportive family and community norms. Behaviours during the COVID-19 pandemic further highlight these findings and provide a setting for positive joint experiences. Based on these findings, programmes and policies to promote AT that emphasize parental support and joint parent and adolescent AT and PA are recommended. Employing social networks and emphasis on opportunities for peer-to-peer communication may also serve as an opportunity to promote AT and PA among adolescents. Finally, these findings support improvements to the built environment, in particular in light of the renewed awareness of the importance of AT facilities during the COVID-19 pandemic.

## Appendix A: Interview Guides on Adolescent Active Travel

### 1. Interview Guide for Adolescents

**Part 1: Review of daily trips**

Please tell me about your daily trips on a typical weekday, before COVID-19. By trip I mean how you get from place to place using different modes of transport including walking, cycling or e-cycling, vehicle, bus, etc. This may include a trip that starts and ends at the same spot, such as walking the dog in your neighbourhood.

Fill out the trip diary together (Table 1).

- Now that we reviewed the trips on a typical day, do you think some of the vehicle trips could be made on foot? How about using a bicycle? What would help you to change your mode of travel?
- Now during COVID-19 tell me about your daily trips? What is similar or different?

**Part 2: Motivations for Active Travel**

Please tell me a bit more about your choices for travel, in particular walking and bicycling.

What makes you choose to walk or bike to a destination?

*(Potential probing questions)*

- Are parents or other adults available to drive you to destinations?
- Is there access to public transport, is it convenient, pleasant?
- Do your parents tell you to walk or bike instead of travel by car? Do they tell you to use the bus?
- Do you walk or bike with your parents to different destinations?
- Do your friends walk and bike to meet or go to destinations?
- Are you in a social network with friends (WhatsApp, Instagram, etc.)? Does travel, walking, or bicycling come up in the groups?
- In your community do you see walkers/cyclists?
- Do you enjoy walking, bicycling? Why? Why not?
- What types of destinations do you travel to on foot, by bike, by e-bike?
- Are there any programmes in your community/school to encourage walking or cycling?
- Is there any traffic in your community? Does it affect mode choice?
- What are the benefits of e-bike vs. walking or cycling?

What prevents you from choosing to walk or bike to a destination?

*(Potential probing questions)*

- When are you less likely to choose walking or cycling?
- Are there cases parents don’t allow you to walk or cycle?
- Are there locations or hours at which you do not walk or cycle?
- Do you feel safe walking? In your opinion, do the drivers in your community give you the right of way?
- Are there areas that are not safe?
- Do your parents discuss safety with you related to walking, cycling, e-cycling?
- How is it different walking alone or with peers? Cycling?
- What don’t you like about walking or cycling?
- Do you think there are differences between boys and girls on this issue?

**Part 3: Physical Activity and Active Travel**

Do you do physical activity (PA) or sports? If so, what kinds of PA and when?

*(Potential probing questions)*

- Is it common to engage in PA with friends? How about Parents?
- How do you feel when you do PA/play sports?
- Do you consider walking to be a type of PA? Biking? E-biking? When and under which conditions?
- How do you feel after a walk or bike ride?
- Do you walk or bike with friends for PA?
- Do you walk or bike with parents for PA?
- Do you consider PA and sports to be similar?
- During COVID-19 is there any change in your PA? Type, frequency, intensity, setting?

**Part 4: Recommendations to Increase Active Travel**

If you could recommend methods to increase walking and bicycling among adolescents your age, what do you think is important?

*(Potential probing questions)*

- What would help you to walk or ride more?
- What about your friends?
- Is there any way to improve the experience for you?

**Part 5: Demographics**

- Gender
- Residence
- Age
- Grade
- Bicycle OwnershipNumber of times a week you did 30 minutes or more of PA (details provided)
- Number of times a week you walked or biked for travel for more than 10 minutes (details provided)
- Distance to school—up to 2 km, more than 2 km (in the town of residence), outside of town

**Table:** Trip Diary

#	Destination?	How did you get there?	Who were you with?	How long did it take?	Who chose this mode of travel?	Why did you choose this mode?
1	School Educational Activity Extra-Curricular Friend Shopping Park/Sports Center Public Transit Other	On foot By Bicycle By E-Bike Electric Scooter Vehicle Bus/Light Rail Train Motorcycle Other	Alone With an adult With an older sibling With a younger sibling With a friend With a group of friends	Time XX:XX- XX:XX Or Number of Minutes	I did My parent/s My friend/s Other	Comfort Habit Parents asked Friends asked Other
2	School Educational Activity Extra-Curricular Friend Shopping Park/Sports Center Public Transit Other	On foot By Bicycle By E-Bike Electric Scooter Vehicle Bus/Light Rail Train Motorcycle Other	Alone With an adult With an older sibling With a younger sibling With a friend With a group of friends	Time XX:XX- XX:XX Or Number of Minutes	I did My parent/s My friend/s Other	Comfort Habit Parents asked Friends asked Other
3	School Educational Activity Extra-Curricular Friend Shopping Park/Sports Center Public Transit Other	On foot By Bicycle By E-Bike Electric Scooter Vehicle Bus/Light Rail Train Motorcycle Other	Alone With an adult With an older sibling With a younger sibling With a friend With a group of friends	Time XX:XX- XX:XX Or Number of Minutes	I did My parent/s My friend/s Other	Comfort Habit Parents asked Friends asked Other
Repeated for additional trips						

### 2. Interview Guide for Parents

**Part 1: Adolescent Travel Modes**

Please tell us about your son/daughter daily trips. By trip I mean how you get from place to place using different modes of transport including walking, cycling or e-cycling, vehicle, bus, etc. This may include a trip that starts and ends at the same spot, such as walking the dog in your neighbourhood.

During a typical week when your son/daughter goes to school, extra-curricular activities, friends, shopping, and other trips how does he/she usually travel?

How are your son/daughter’s trips usually distributed across the different modes of travel?

**Table:**

Mode	Most trips	Some Trips	Few Trips	No Trips
Walking				
Cycling				
E-cycling				
Electric Scooter				
Personal Vehicle				
Bus/ Light Rail				
Train				
Pupil Transport				
Other				

For those modes that were listed as no trips, why is that mode not used?

How is the situation you described similar or different now, during COVID-19? Has there been an increase or decrease in any particular mode of travel? Do you have any new rules in place regarding travel during COVID-19?

**Part 2: Active Travel and Physical Activity**

Now I would like to hear from you further about you and your son/daughter’s travel.

Do you like to walk or cycle to different destinations? What makes you choose to walk or bike to a destination?

*(Potential probing questions)*

- Do you walk or cycle to different destinations in your town/city?
- Do you walk/cycle alone? With your children?
- Do you use public transport?

Do you ever ask your son/daughter to walk, cycle, use public transport to destinations? When, Why?

*(Potential probing questions)*

- Are you available to provide rides to your son/daughter?
- Do you also encourage walking/cycling when there is a vehicle available?
- Are there situations in which you don’t allow your son/daughter to walk/bike to destinations?

In your opinion, are there any benefits to your son/daughter walking or biking?

*(Potential probing questions)*

- What are the benefits for your son/daughter?
- What are the benefits for you? For your family?

In your opinion, are there any disadvantages to your son/daughter walking or biking?

- What are the disadvantages for your son/daughter?
- What are the disadvantages for you? For your family?

Do you do physical activity (PA) or sports? If so, what kinds of PA and when?

**Part 3: Facilitators and Barriers to Active Travel**

What are some of the conditions that facilitate walking and cycling?

What are some of the barriers to walking and cycling?

*(Potential probing questions)*

- In your opinion, is your neighbourhood/town pleasant for walking? Cycling?
- In your opinion, is your neighbourhood/town safe for walking? Cycling?
- Do the facilities (paths/equipment) encourage walking? Cycling?
- Is there access to public transport, is it convenient, pleasant?
- Do you think that it is common among your son/daughter’s peers to walk or cycle?
- Does your son/daughter walk or cycle together with peers to different destinations?
- In your community is walking and cycling common?
- Are there any programmes in your community or local schools to encourage walking or cycling?
- Is there any traffic in your community? Does it affect mode choice?
- How do you suggest improving the walking/cycling experience in your community?

**Part 4: Recommendations to Increase Active Travel**

If you could recommend methods to increase walking and bicycling among adolescents, what do you think is important?

*(Potential probing questions)*

- What would help parents of adolescents to encourage walking and cycling?
- Is there any way to improve the experience for you? For your family?

**Part 5: Demographics**

- Gender
- Residence
- Age
- Vehicle Ownership
- Number of times a week you did 30 minutes or more of PA (details provided)
- Number of times a week you walked or biked for travel for more than 10 minutes (details provided)
- Education level: High school graduate, Post high school certification, Academic – Bachelors, Academic – Masters or above
- Household income: In comparison to average household income based on data from the Central Bureau of Statistics 2020, is your household income average, above average, below average
